# Quality Versus Quantity of Life: Beyond the Dichotomy

**DOI:** 10.1089/pmr.2022.0058

**Published:** 2023-03-17

**Authors:** Ariel Dempsey, John Mulder

**Affiliations:** ^1^ Faculty of Theology and Religion, University of Oxford, Oxford, United Kingdom.; ^2^ Department of Family Medicine, Division of Palliative Medicine, Michigan State University College of Human Medicine, Grand Rapids, Michigan, USA; Trillium Institute, Spring Lake, Michigan, USA.

**Keywords:** decision making, end-of-life care, goals of care, palliative care, physician patient communication, quality of life

## Abstract

A restrictive and dichotomous question has become the primary approach in many goals of care discussions. Is the primary goal of care *quantity* of life through aggressive therapy or *quality* of life through comfort care and hospice? Even though many health care providers recognize that quality vs quantity of life (QvQ) is a false dichotomy, in practice QvQ underlies many goals of care discussions and can negatively impact patient care. This article offers strategies for assessing patients at the end of life, presenting a first-line conversation process that can support a range of treatment options as well as a diversity of dynamic patient values. Based on decades of experience in palliative care and a review of relevant literature, we recommend four practical questions to serve as values “vital signs,” monitoring dynamic notions of quality of life and harmonizing patient values with treatment options.

## Introduction

“It is essential, here as elsewhere, to abandon old habits of Gleichschaltung, the deeply ingrained worship of tidy-looking dichotomies.”

—J.L. Austin^1^

Mike lay in a hospital bed—a ventilator down his throat, feeding tube in his side, intravenous cannula in his arm, catheter in his bladder. He had end-stage amyotrophic lateral sclerosis (ALS). Unable to move, unable to communicate, no hope for recovery.

As seen in the case of Mike, advances in scientific knowledge and medical technology that lengthen life can also create a burden of suffering. How can we better address issues of quality and quantity of life with our patients? There is much research to support importance of goals of care conversations,^2–9^ yet studies show that goals of care are inadequately addressed.^2,6,10–20^

Conversations with physicians, colleagues, and patients reveal that a dichotomous way of talking about quality versus quantity of life (QvQ) underlies many goals of care discussions and impacts patient care.^10,16,21–33^ A majority of the literature on quality and quantity of life are studies of patient decision preferences in oncology,^25,28,34–36^ time trade-off utility studies,^37,38^ and studies of patient preferences in end-of-life care.^24,31,39,40^ Some literature comments on how medicine has, at times, pressed for quantity of life at the expense of quality of life.^22,41–44^ Very few articles comment on how QvQ is used in practice when discussing treatment options and goals of care.^21,25,45^ Even fewer reflect on potential strengths and dangers of QvQ.^21^

In discussions with terminally ill patients, it is not uncommon for a physician to ask a patient whether their primary goal is quantity of life through aggressive therapy or quality of life through comfort care and hospice. This dichotomy can become a source of conflict for patients, families, physicians, and health care workers. Furthermore, it reinforces negative stereotypes of hospice and palliative care.

**FIG. 1.**
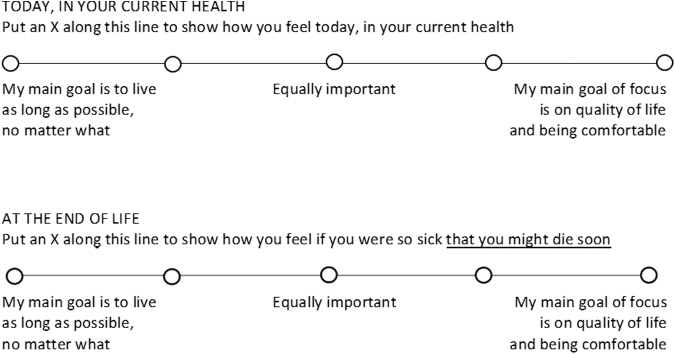
“Treatment Preference Goals of Care” form. Example of advance care planning document used in a hospital system.^46^

Although many physicians recognize that QvQ is a false dichotomy, in practice QvQ drives many goals of care discussions. For example, at one of the larger health systems in the Midwest, patients complete a “Treatment Preference Goals of Care” form (Fig. 1).^46^ Many hospitals use similar forms to guide discussion of treatment plans.

As seen earlier, quality of life and quantity of life are frequently presented as opposites. For patients with all types of medical conditions, one quality of life “length of life” is set against all other qualities of life in a relationship of conflict and competition.

Studies show that patient preferences are affected by how survival probabilities are framed, side effects are presented, and questions are asked.^10,15,25,26,47,48^ Our language can impact patient choices and simplistic questions fail to guide patients in decision-making processes that truly reflect their values:

- Do you want to give up?
- Do you want to fight?
- Do you want your goal of care to be quality or quantity of life?

QvQ was a tool created to initiate conversations about goals of care, but the restrictive and dichotomous nature of QvQ make it problematic when used on its own, as a first-line approach to goals of care discussions and primary determinant of care. Studies show that patients desire a balance between quality and quantity in their goals of care.^31^ As demonstrated in the following case study, we remind providers to seek this balance by situating QvQ within a larger more inclusive framework of Values-Treatments Harmony.

## Case Study: Values-Treatments Harmony

Mike was a man in his forties presenting to the ALS clinic with a new diagnosis of ALS. As he shuffled down the hallway for his first visit, he passed another ALS patient advanced in her illness. She sat motionless in an electric wheelchair with a respiratory assist machine strapped to her back, eye gaze computer on her right, and bag of nutrients at her left. Mike was shaken by what he saw. When he met his physician, he said, “Doctor, don't EVER let me get to that point! That's not quality of life.” His physician assured him that no intervention would be done without Mike's authority. He asked Mike about his understanding of his condition and treatment plan and explained that today was the beginning of an ongoing discussion about his wishes for the management of his condition.

A few weeks later, Mike was struggling with ambulation. His doctor suggested trial of an electric wheelchair. When he returned to clinic two weeks later with his family, Mike was ecstatic about his newfound mobility. His physician noted significant weight loss due to dysphagia. Mindful of Mike's philosophy of nonintervention, Mike's physician asked him about his current enjoyment of life. Mike replied that he was generally comfortable and enjoying time with loved ones. His physician noted that the current weight trajectory would shorten Mike's life, but that nutritional supplementation could prolong it. Reassured that he could discontinue anytime, Mike decided to proceed with placement of a feeding tube. He tolerated it well and was pleased.

Some weeks later, Mike noticed persistent fatigue interfering with day-to-day life. He and his family scheduled an appointment. Evaluation revealed that low oxygen while sleeping was impacting his vitality. His physician offered ventilatory support: not a machine that would breathe for him, but one that would increase pressure for his breathing mechanism, now compromised due to weakness of his chest muscles. Reassured that this could be stopped whenever he wanted, Mike agreed to a trial. He was delighted—resting better, feeling “more alive” and sharing meaningful moments with loved ones. In some ways, he resembled the woman he saw on the first day at the ALS clinic. He realized that although he valued the ability to walk, eat, and breathe on his own, what he valued more was relationship with his family, and he was grateful for medical interventions that made these relationships possible.

Some months later, Mike's communication became compromised. He could no longer interact except through nods and unintelligible sounds. Soon after, Mike was hospitalized for aspiration pneumonia. Mike lay in a hospital bed—a ventilator down his throat, feeding tube in his side, IV in his arm, catheter in his bladder. He indicated that it was time to allow the disease to take its natural course and requested discontinuation of nutritional support, in agreement with his family. Peacefully, he died a week later with his family by his side.

QvQ narratives often begin with the last paragraph of Mike's story. A goals of care meeting is called late in the course of illness^7,18^ and discussion contrasts quantity of life through aggressive therapy or quality of life through comfort care and hospice.

The broader story of Mike illustrates how this dichotomy is too small a framework for dynamic realities of end-of-life care. Mike's early response to the idea of medical intervention was, “Doctor, don't EVER let me get to that point. That's not quality of life.” But as Mike's illness evolved, so did his concept of quality of life.^49^ The electric wheelchair, feeding tube, and ventilatory assist device lengthened his life and improved qualities of life that he valued.^50^ Had the physician adopted Mike's initial assertion as determinant of preference for quality over quantity, and avoided further discussions about medically aggressive life-sustaining options, Mike would have been deprived of the richness of his extended life.^27^ Mike's physician guided him so as to extend life longer than the normal disease trajectory, while allowing that extension of life to have meaning and purpose.^49,51,52^

Let us pull some examples from the case of Mike showing further how presenting goals of care as a QvQ dichotomy can be confusing for patients. First, comfort measures improved Mike's quality of life and extended his life. Both quality and quantity were his goals. The placement of the feeding tube, for example, was done with the goal to prolong his life. The ventilatory assist was done with the goal to improve his quality of life. This quality of life intervention improved some qualities of life and decreased other qualities of life, for example, increasing reliance upon technology, which he had previously viewed as unacceptable.

Upon accepting the ventilatory assist, he resembled the woman he saw at the first day of the ALS clinic, yet he felt that this intervention was worth it because it allowed him something he valued more—meaningful connection with his family. Moreover, Mike experienced positive life experiences with his family, which arose because of his disease journey. His physician did not ask about Mike's preferences for care by labeling one choice “quantity” and one choice “quality.” Instead, his physician helped Mike's harmonize treatment options with Mike's values.

Imagine if Mike thought, as many patients do, that if he chose quantity of life through aggressive therapy, he would be abandoned with regard to comfort care and that if he chose quality of life through comfort care he would be “giving up” and hastening death. Presenting the choice of quality or quantity, they may choose quantity and order to pursue unnecessary and futile treatments. Or imagine if Mike had developed pneumonia early in his disease progression and confusion about what his designated choice “quality of life” meant prevented him from receiving temporary intubation and antibiotics that would extend life and allow him those meaningful last six months with his loved ones.

Mike's story illustrates that we need not limit our patients to choosing between conflicting options of “living longer” or “living better.” We have opportunity for deeper more personalized engagement, exploring values that matter to our patients, establishing goals consistent with those values, and outlining plans of care that best achieve those goals. We call this Values-Treatments Harmony.

## Strategies for Conversations: Values-Treatments Harmony

Many physicians feel underequipped for goals of care conversations.^2,15,20,53–55^ Based on decades of experience in palliative care and a review of literature on goals of care discussions, we recommend four practical questions to serve as values “vital signs,” monitoring dynamic notions of quality of life.

**1. What do you understand about your disease?**^2,13,15,28,54,56–58^ Clinicians can explore whether the patient understands the nature of the disease, what to expect as it progresses, potential for cure (or remission), and prognosis.
**2. What is your understanding of the plan?**^13,15,56–58^ A patient's knowledge of the current plan and why it is being implemented is important. This question helps physicians uncover gaps in patients' understanding of their care.
**3. What matters to you?**^2,4,39,48,54,56–61^ Identifying patient values is a critical step in this process. If they have a limited life expectancy this question can be used to identify achievable goals that they can accomplish before death. Relevant questions include the following: “What brings you joy and peace?” “What relationships are important?” “What makes you feel fulfilled and gives you a sense of purpose?” What is illuminating about the case of Mike is that his answer to the question, “What matters to you?” changed over time, adjusted as he engaged questions about next steps in his treatment. Patient values are embodied in shifting contexts; they can be as dynamic as their health conditions, and it is important to continuously monitor them.^25,52,62,63^
**4. Are the plans currently in place helping you to achieve what is important to you, or standing in the way of your goals?**^2,3,13,15,39,54,56–58,64^ This question connects the plan of care to the patient's values and clarifies direction of care. It emphasizes the present—taking one day, one step of treatment, at time.

Once these open-ended questions^48,57,60^ have been engaged, the clinician and treatment team share the responsibility of guiding the patients toward a practical plan that resonates with patient values.^12,48,54,57^ For example, it may include dialogue such as “based on what you've told me about what is important to you and what you'd like to accomplish with your time left, I would recommend that you consider___as a plan consistent with your values and wishes.”^4,54^

This process requires revisiting values and management strategy throughout the course of illness.^48,54,62^ One systemic challenge to longitudinal discussions is the fact that patient care is often provided by multiple people in multiple settings. Poor communication and inadequately integrated information systems can result in patients having to begin the conversation anew at each visit. Although sometimes revisiting their history can bring fresh perspective and new insight it can also be exasperating and exhausting for patients if they feel that previous conversations have not been recorded, shared, or respected.

Similar to vital signs that are tracked by rotating teams of providers, these questions can help track dynamic changes in a patient's condition through time and help physicians optimize care. Monitored frequently and checked repeatedly, they can be evaluated for trends, and care can be adjusted accordingly. As vitals are understood in reference to patient's baselines, so these conversations should reflect back and build upon what a patient has already shared. Tracking values such as vitals, a physician can partner with their patient in harmonizing values and treatment options.

## Beyond the Dichotomy

The story of Mike raises doubts about the adequacy of QvQ as a leading strategy for goals of care conversations. Many patients really do think they are choosing between “living longer” or “living better.” Several points of observation from clinical experience underscore how presenting quality of life and quantity of life as competitive alternatives can be confusing for our patients and for families/caregivers trying to navigate difficult decisions on behalf of the their loved one:

- “Comfort measures” may have the capacity to extend life.^7,65^
- Some things we do to improve quality of life can create an alternative burden of suffering and decrease quality of life in another sense.^66,67^
- Many interventional treatment measures intended to extend life can improve quality of life in unanticipated ways.^68,69^
- Extending life may be essential to a patient's definition of quality of life.^64^
- Some interventions intended to extend life can actually shorten life.^32,33,66^
- Patients who encounter challenging or tragic health events will often report positive life experiences that arose because of their disease journey.^70–72^
- A patient can be asked about their wishes regarding treatments, cardiopulmonary resuscitation, ventilators, and feeding tubes without labeling one choice quantity and one choice quality.^62,73^
- Language of “quality” versus “quantity” can force a dichotomous choice and bias patient decision.^10,21^

In some circumstances, length of life can compete with other values a patient considers essential to their quality of life. In oncology, for example, the prevalence of situations in which the QvQ dichotomy may be relevant is higher than in other illness populations. The emotional devastation of a cancer diagnosis can be intense, and the desire to live longer juxtaposed with the frequent symptomatic challenges with cancer-directed therapies can generate significant angst in patients and families, particularly if options are presented as “do you want the possibility of living longer (quantity), or just remain comfortable (quality)?” In diseases that have no cure, but for which treatments are available decision making becomes complex. Many patients desire to live longer AND to avoid suffering.

This once again emphasizes the importance of clinicians helping patients focus on the values most meaningful to them. Each disease, and each person, has their own unique illness trajectory and valued qualities of life to reconcile. For this reason, QvQ may be a useful subquestion within a larger framework of Values-Treatments Harmony. However, as first-line question QvQ can be problematic because it restricts the complex realities of medical situations. In the words of the J.L. Austin epigraph, QvQ is a “tidy-looking dichotomy.” We need to expand our approach in ways that allow us to be more attentive to persons as individuals whose experiences cannot be sorted into simple categories of quality or quantity.

This narrative review is just a starting point. Moving beyond the QvQ dichotomy and subordinating it within Values Treatments Harmony may have implications for health care systems improvements. For example, Medicare patients are asked to choose between quality and quantity of life when they elect for hospice benefits. Hospice regulations specifically state, “An individual must waive all rights to Medicare payments for…any Medicare services that are related to the treatment of the terminal condition for which hospice care was elected…”^74^ What is known to be a false dichotomy becomes very “real” to these patients as the system is written to make quality and quantity of life competing priorities.

It is always challenging to invoke systemic cultural change, but we feel that such change is possible in the case of the QvQ paradigm. The beginning of this transformation will be in how providers communicate with patients and families. Harmonizing clinical reality and treatment options with a diversity of patient values will foster less emphasis on a QvQ conversation. To that end, we propose that communication curricula in medical schools and postgraduate training programs begin to integrate a paradigm of conversation that emphasizes the centrality of patient values and diminish the focus on traditional QvQ language. In addition, future studies can be developed to assess the impact of the QvQ dichotomy on patients, caregivers, health care providers, and health care systems and trials to evaluate the effectiveness of the four suggested questions.

## Abbreviations Used

ALS: amyotrophic lateral sclerosis
QvQ: quality versus quantity of life
